# Case Report: S100-negative primary anorectal melanoma with elevated Ki67: a case for biology-driven radical resection despite early radiological staging

**DOI:** 10.3389/fonc.2025.1682861

**Published:** 2025-12-11

**Authors:** Xiangxiang Ren, Tianhao Xie, Litao Liu, Xiaoshi Jin, Wei Ma, Jing Sun, Meng Zhang

**Affiliations:** 1 Department of General Surgery, Affiliated Hospital of Hebei University, Baoding, Hebei, China; 2 Department of Dermatology, Affiliated Hospital of Hebei University, Baoding, Hebei, China

**Keywords:** primary anorectal melanoma, Ki67 proliferation index, abdominoperineal resection, immunohistochemistry, S100-negative, treatment decision-making

## Abstract

**Background:**

Primary anorectal melanoma (PARM) is a rare and highly aggressive malignancy. Diagnosis is often challenging due to non-specific symptoms and potential S100 negativity on immunohistochemistry (IHC), while optimal surgical management remains debated.

**Case presentation:**

A 67-year-old woman presented with hematochezia, tenesmus, and overflow pseudodiarrhea. Clinical examination and MRI identified a 2.0 cm ulcerated rectal mass, staged as cT2N0. Initial biopsy revealed atypical melanocytic proliferation. Definitive IHC of the resection specimen confirmed melanoma, showing S100 negativity but positivity for HMB45, Melan-A, and SOX10. A notably high Ki67 proliferation index was observed, escalating from 30% in the biopsy to 60% in the resected tumor. Despite the early radiological stage (cT2N0), the high Ki67 index indicated aggressive tumor biology, prompting a multidisciplinary team to recommend abdominoperineal resection (APR). Adjuvant chemotherapy with temozolomide and cisplatin was administered postoperatively. The patient remained disease-free at 24-month follow-up.

**Conclusion:**

This case highlights that S100 negativity does not preclude a melanoma diagnosis when supported by other specific melanocytic markers. Furthermore, a markedly elevated Ki67 index may identify biologically aggressive PARM tumors that could benefit from radical resection, even in the context of early radiological staging. Biology-driven surgical decision-making, complemented by adjuvant therapy, may improve outcomes in this high-risk subset of patients, though larger prospective studies are needed for validation.

## 1 Introduction

Primary anorectal melanoma (PARM) accounts for <1% of all melanomas and 0.1–0.5% of anorectal malignancies (1). Its prognosis is poor (5-year survival: 10–20%), often due to delayed diagnosis and early metastasis (2). Patients typically present with symptoms mimicking benign anorectal disorders (hemorrhoids, adenomas), and histopathological diagnosis is complicated by morphological heterogeneity and variable immunohistochemical (IHC) expression (2). Although S100 protein is highly sensitive for melanoma, it is negative in 3–8% of mucosal melanomas and up to 24% of anorectal melanomas, necessitating reliance on other IHC markers for diagnosis (3–6). The Ki67 proliferation index is emerging as a critical prognosticator, with values >20% associated with reduced survival in mucosal melanomas (7).

Current management of primary anorectal melanoma is challenging, with radical surgery (abdominoperineal resection, APR) and wide local excision (WLE) representing the main surgical options. However, the optimal strategy remains debated due to the rarity of the disease and a lack of high-level evidence (8). A recent systematic review and meta-analysis by Smith et al. (2020) reported no significant overall survival benefit for APR over WLE, while highlighting the increased morbidity associated with APR, which has led to a growing consensus favoring organ-preserving WLE when feasible (8). In contrast, other studies, such as a meta-analysis by Temperley et al. (2022), emphasize the significantly reduced local recurrence rates with radical surgery (9). Current National Comprehensive Cancer Network (NCCN) guidelines include local excision as a potential option for selected early-stage PARMs, but they explicitly acknowledge the limited evidence supporting this approach (10). Diagnosis, staging, and prognostication rely on histopathological confirmation (noting the potential S100 negativity in a subset of cases), imaging, and prognostic markers such as the Ki67 proliferation index. We report a case of localized PARM (cT2N0) where a markedly elevated Ki67 index (60%) prompted serious consideration of APR despite the clinical stage, illustrating the critical role of tumor biology in guiding surgical decision-making for this complex disease.

## 2 Case report

### 2.1 Clinical presentation

A 67-year-old postmenopausal woman presented with a 6-month history of progressive hematochezia, tenesmus, and overflow pseudodiarrhea (Bristol type 5–6 stools). She had a past medical history of hypertension controlled with amlodipine, and type 2 diabetes managed with metformin. There was no family history of melanoma or other malignancies. The patient was a non-smoker and denied alcohol use. Psychosocial assessment revealed no significant stressors or occupational exposures. Digital rectal examination revealed a firm, non-tender 2.0 cm ulcerated mass 2 cm proximal to the dentate line.

### 2.2 Diagnostic workup

**Figure 1:**
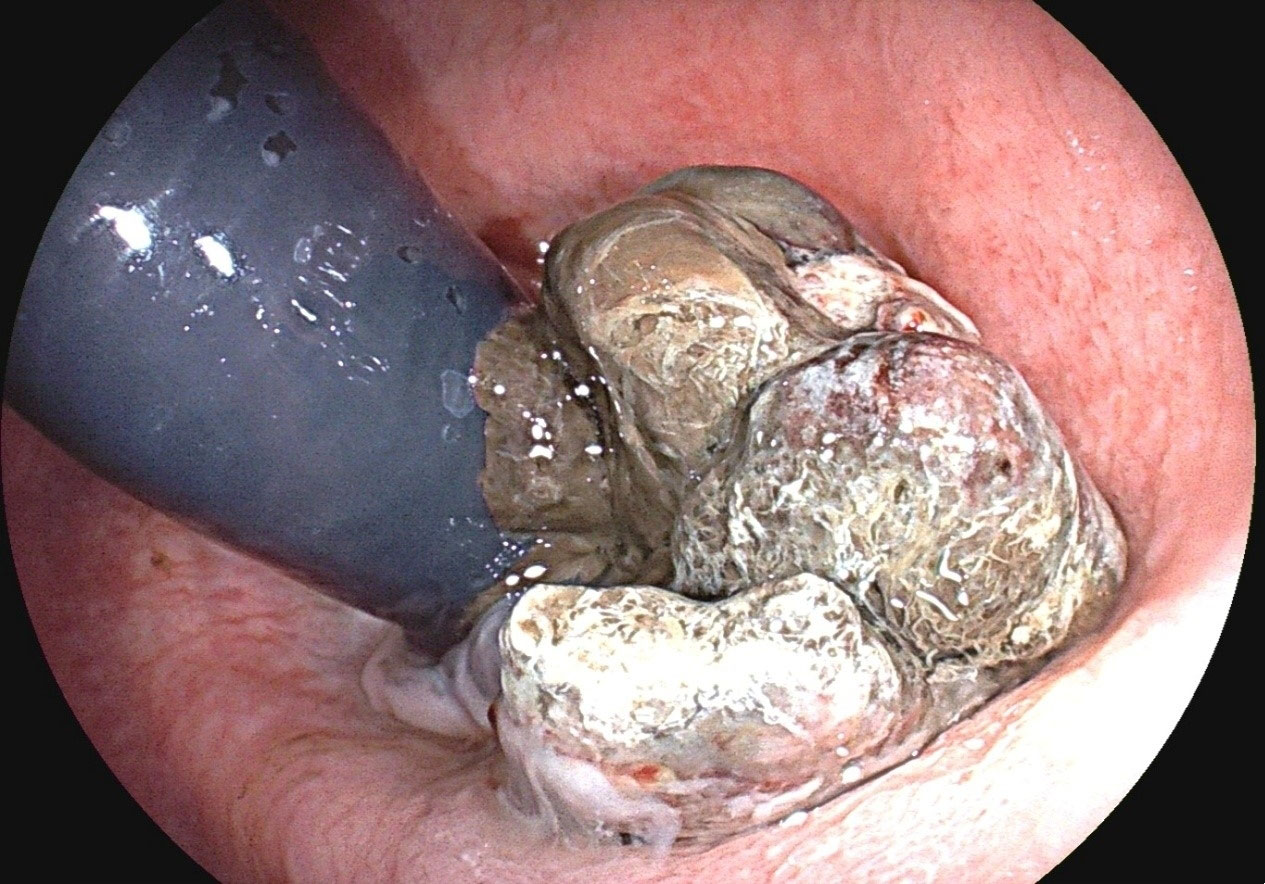
Sigmoidoscopy revealing an ulcerated, pigmented, friable mass in the distal rectum.

**Figure 2:**
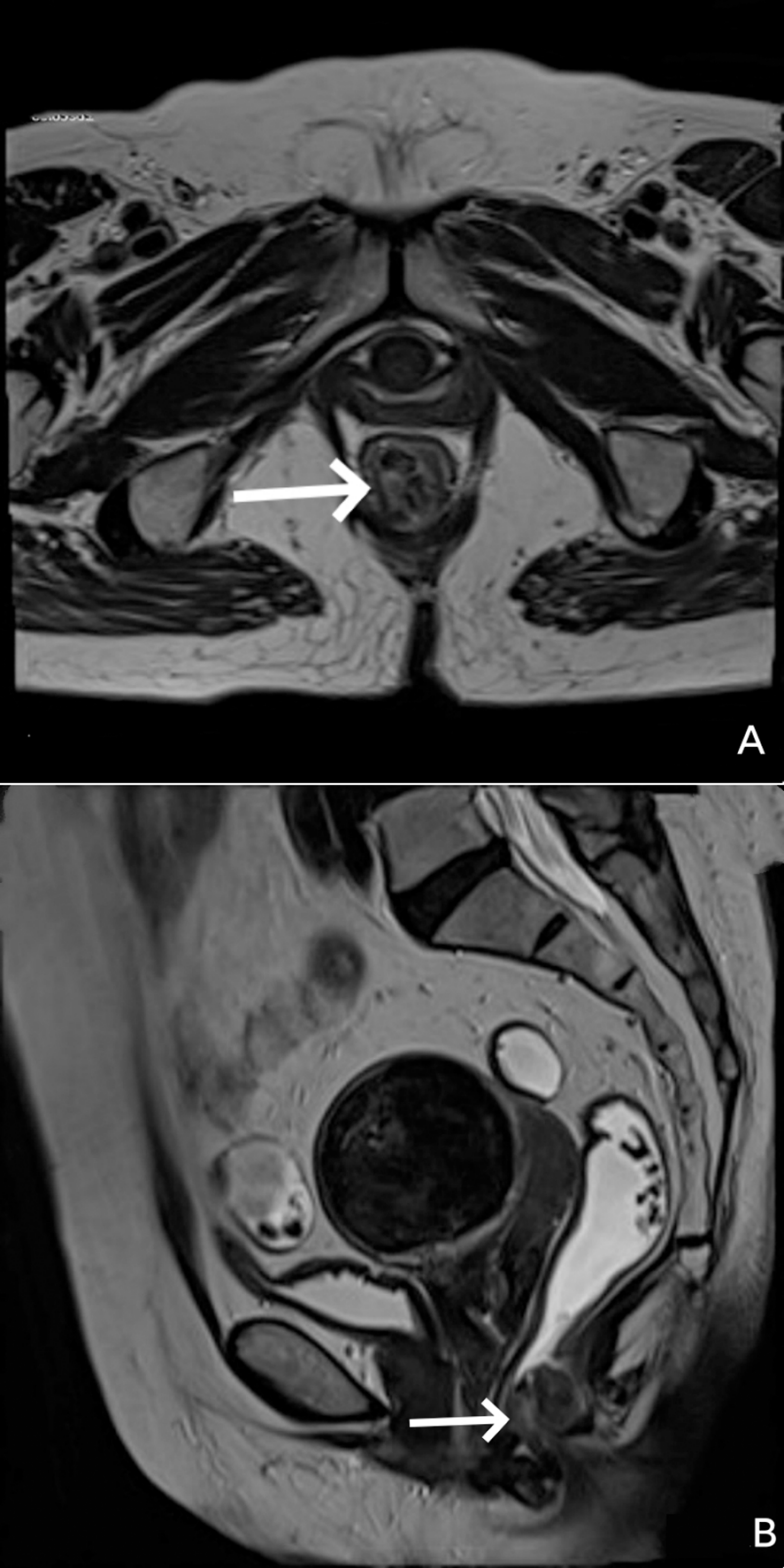
**(A)** Axial and **(B)** Sagittal T2-weighted MRI images demonstrating a T2-hyperintense tumor confined to the rectal wall.

Sigmoidoscopy revealed an ulcerated, pigmented, friable mass in the distal rectum with active bleeding (**Figure 1**). Pelvic MRI demonstrated a T2-hyperintense tumor confined to the rectal wall without mesorectal lymphadenopathy or extramural invasion, staged clinically as cT2N0 (**Figures 2A, B**). Initial biopsy demonstrated atypical melanocytic proliferation. Metastatic workup, including comprehensive dermatological/ophthalmological examination and contrast-enhanced CT of the chest/abdomen/pelvis, identified no primary cutaneous/mucosal lesions or distant metastases.

### 2.3 Histopathology and molecular analysis

**Figure 3:**
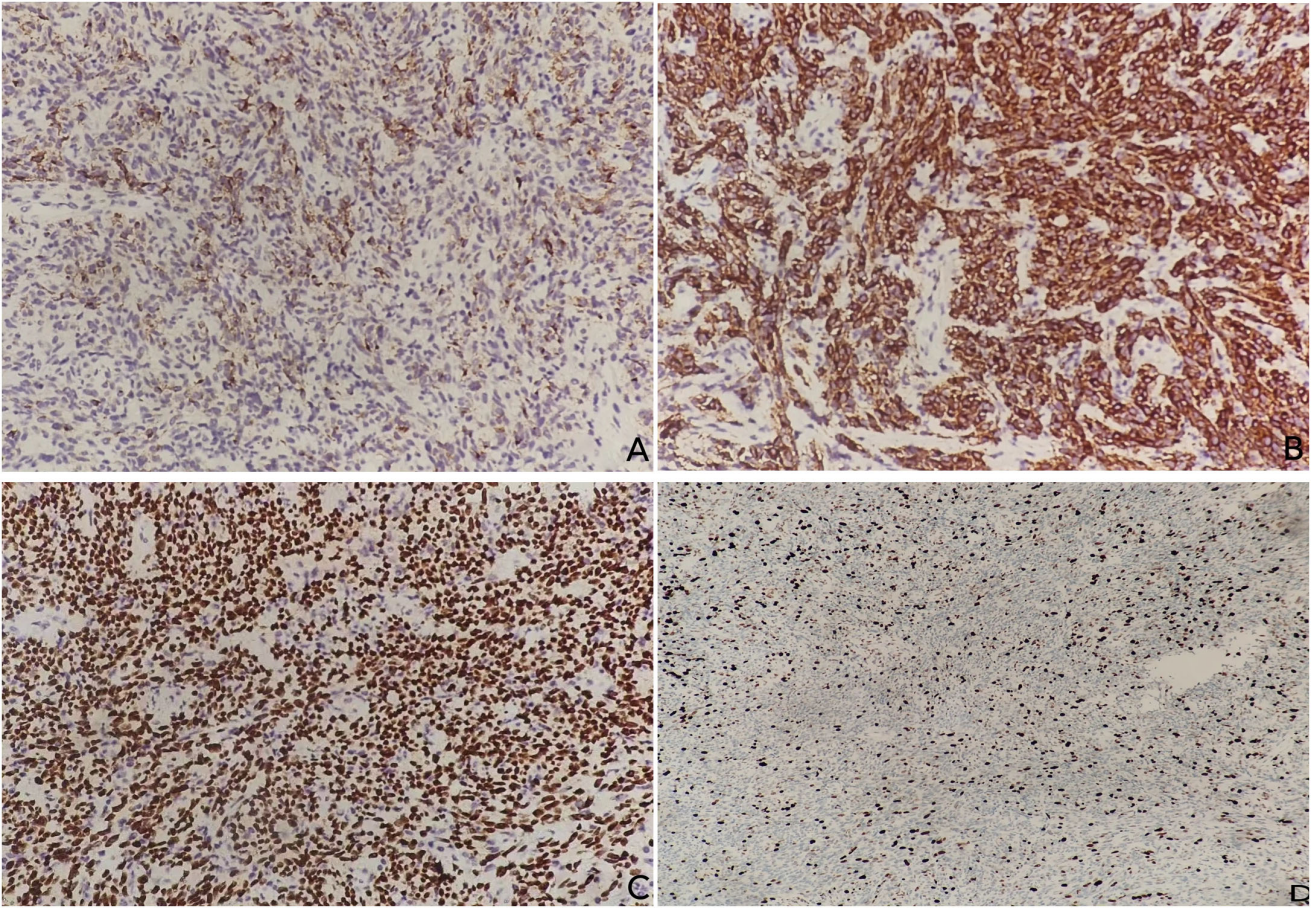
Immunohistochemical analysis of the resection specimen. **(A)** Diffuse positivity for HMB45. **(B)** Diffuse positivity for Melan-A. **(C)** Strong nuclear positivity for SOX10. **(D)** Ki67 immunostaining showing a high proliferation index of 60%.

Initial biopsy demonstrated atypical melanocytic proliferation. Multiple biopsies were obtained from different regions of the ulcerated mass to account for potential tumor heterogeneity. Histopathology of the surgical resection specimen revealed malignant epithelioid and spindle cells with pleomorphic nuclei, prominent nucleoli, and cytoplasmic melanin deposition (**Supplementary Figure S1A, B**). Immunohistochemistry (IHC) was diffusely positive for HMB45, Melan-A, and SOX10 (**Figures 3A–C**; **Supplementary Figure S3A–C**), but consistently negative for S100. The Ki67 proliferation index was 60% in the resection specimen(**Figure 3D**) (compared to 30% in the pre-operative biopsy). Molecular testing confirmed wild-type status for BRAF, V600E, and NRAS.

### 2.4 Management and follow-up

The chronological sequence of key diagnostic, therapeutic, and surveillance events is summarized in **Table 1**. Multidisciplinary tumor board review recommended definitive surgical management. Despite cT2N0 staging, the elevated pre-operative Ki67 index (30%) and S100 negativity were indicative of high biological aggressiveness, supporting abdominoperineal resection over sphincter-sparing options. The patient underwent laparoscopic APR with total mesorectal excision, achieving R0 resection (final pathological staging: pT2N0). Adjuvant chemotherapy with temozolomide (200 mg/m²/day, days 1–5) and cisplatin (25 mg/m²/day, days 1–5) was administered every 21 days for 6 cycles. The treatment was well tolerated, with 100% adherence and no grade 3–4 adverse events reported; only mild nausea and fatigue were noted, which were managed supportively.

**Table 1:** Timeline of key diagnostic, therapeutic, and surveillance events.

Time point	Event	Key findings/outcomes
Day 0	Initial Presentation	Hematochezia, tenesmus, pseudodiarrhea
Day 1	Sigmoidoscopy & Biopsy	Ulcerated rectal mass; Atypical melanocytic proliferation (Ki67 30%)
Day 7	Pelvic MRI	cT2N0 staging
Day 14	Metastatic Workup (CT)	No distant metastases
Week 3	Multidisciplinary Decision	Recommended APR based on high Ki67 and S100 negativity
Week 4	Surgery (APR)	R0 resection, pT2N0; Ki67 60% on final pathology
Month 1-4	Adjuvant Chemotherapy	6 cycles of Temozolomide + Cisplatin; well-tolerated
6, 12, 18, 24 months	Surveillance	No evidence of recurrence on clinical exam and imaging (CT/PET-CT)

Postoperative surveillance included quarterly clinical assessments and serial imaging. Surveillance CT at 6 and 12 months showed no recurrence, and 18-month PET-CT confirmed no evidence of distant metastases. At the 24-month follow-up, the patient remained disease-free. She reported satisfactory stoma function and acceptable quality of life during follow-up visits, with no signs of depression or anxiety related to the surgical outcome.

## 3 Discussion

Primary anorectal melanoma represents a therapeutic challenge due to its rarity, diagnostic complexity, and poor prognosis (median overall survival: 12–24 months) (11). This case highlights critical limitations in two clinical paradigms: 1) diagnostic overreliance on S100 protein expression, and 2) exclusive dependence on radiological staging for surgical planning. We contextualize these findings with contemporary evidence.

### 3.1 Diagnostic pitfalls in S100-negative PARM

PARM typically demonstrates epithelioid or spindle cell morphology with variable melanin deposition, often mimicking gastrointestinal stromal tumor or poorly differentiated carcinoma (12). Although S100 positivity exceeds 90% in cutaneous melanomas (13), its absence in 3–15% of mucosal melanomas and up to 24% of anorectal melanomas creates diagnostic uncertainty (4–6). As observed in our case, S100-negative melanomas frequently retain SOX10 and MITF expression. One proposed mechanism suggests neural crest-derived tumors may downregulate S100 during mucosal microenvironment adaptation (14). SOX10’s consistent nuclear staining pattern (strongly positive in our case) may help maintain diagnostic accuracy when S100 is lost (14). Therefore, suspected PARM specimens should routinely undergo an IHC panel comprising SOX10, HMB45, Melan-A, and MITF to confirm melanocytic differentiation, regardless of S100 status (14, 15).

### 3.2 Ki67 as a prognosticator and surgical decision-modifier

The key innovation in this case’s management strategy was the use of a preoperative Ki67 index exceeding 30% to justify proceeding with radical resection despite clinical staging indicating cT2N0 disease. This decision was grounded in contemporary evidence establishing the significant prognostic value of Ki67 in mucosal melanoma, where the index correlates strongly with disease stage and overall survival, serving as an independent prognostic biomarker (16, 17). Patients exhibiting a Ki67 index ≥40% in other mucosal melanomas frequently present with advanced disease and experience significantly reduced survival (16). This prognostic significance is corroborated specifically in PARM, where a Ki67 index ≥40% or a PCNA index ≥80% independently predicts poorer outcomes (17).

The limitation of MRI in accurately assessing the underlying biological aggressiveness of these tumors is highlighted by findings such as those from Kimura et al. (2020), who reported occult serosal invasion undetected by imaging in 22% of radiologically staged cT2 PARM cases, resulting in pathological upstaging to pT3 (18). The notable discordance observed in our patient between the preoperative biopsy (Ki67 30%) and the final resection specimen (Ki67 60%) underscores several critical considerations: 1) the inherent sampling limitations associated with fragmented biopsies, 2) the significant intratumoral heterogeneity of proliferative activity, and 3) the consequent risk of underestimating the true biological aggressiveness of the tumor when relying solely on local excision techniques.

### 3.3 Radical resection vs. local excision: integrating biomarkers into surgical decision-making

Our illustrative case—featuring a tumor size of 20 mm and a preoperative Ki67 index of 30% that increased to 60% in the resection specimen—exemplifies how biomarker assessment can identify biologically aggressive PARM despite radiologically localized (cT2N0) disease. These findings collectively suggest that the presence of concurrent high-risk features, such as a Ki67 index ≥40% and/or tumor size ≥20 mm, may justify radical resection even when conventional imaging suggests early-stage disease.

While current National Comprehensive Cancer Network guidelines include local excision as a potential option for selected early-stage PARMs, they explicitly acknowledge the limited evidence supporting this approach (10). Our decision to proceed with abdominoperineal resection aligns with emerging oncologic principles emphasizing aggressive local control. This is supported by a recent meta-analysis demonstrating significantly reduced local recurrence rates with radical surgery versus local excision (odds ratio 0.15, 95% CI 0.08–0.28, *p* < 0.00001) in non-metastatic PARM (9). The integration of prognostic biomarkers further guides the escalation of surgical management. Specifically, a Ki67 index ≥40% independently predicts poor prognosis and aggressive tumor biology (17), while a tumor size exceeding 20 mm correlates with increased recurrence risk and worse survival (19), and a Breslow thickness greater than 2 mm portends higher rates of nodal metastasis (19).

It is important to acknowledge that the surgical management of PARM remains controversial. A recent meta-analysis by Jutten et al. (2021) including 1,858 patients concluded that ‘there is no evidence to suggest that a radical primary surgical strategy improves outcomes in ARM. Therefore, given the well-documented morbidity associated with APR, WLE with regular surveillance for local recurrence should be the primary strategy in most patients’ (20). This perspective highlights the need for careful patient selection when considering radical resection. Our case suggests that biomarkers such as Ki67 may help identify the minority of patients who could benefit from more extensive surgery, though this hypothesis requires validation in larger, prospective studies that directly compare outcomes between WLE and APR in patients stratified by biological markers.

### 3.4 Molecular landscape and biomarker-driven adjuvant therapy

PARM exhibits a distinct molecular profile characterized by a notably low frequency of *BRAF* and *NRAS* driver mutations, occurring in less than 10% of cases compared to over 50% in cutaneous melanoma (21). Our patient’s *BRAF V600E/NRAS* wild-type status aligns with this established pattern, thereby precluding first-line targeted therapy options. Given the absence of actionable mutations, adjuvant chemotherapy with temozolomide-cisplatin was selected based on prospective clinical evidence; specifically, a Phase II trial (N = 57) in mucosal melanoma demonstrated significantly improved 2-year disease-free survival with postoperative temozolomide (200 mg/m²/day D1-5) plus cisplatin (25 mg/m²/day D1-5) versus observation alone (36.1% vs. 20.5%; *p* = 0.023) (22). Despite the patient showing no evidence of disease at 18-month follow-up, rigorous long-term surveillance remains imperative due to PARM’s aggressive recurrence dynamics. Approximately 80% of recurrences manifest within 24 months post-resection, with hepatic (45%) and pulmonary (32%) metastases representing the predominant sites of relapse (23), underscoring the necessity for sustained vigilance even after successful initial intervention.

### 3.5 Limitations and unanswered questions

Several limitations and unresolved questions merit consideration in our management approach. First, the spatial heterogeneity of Ki67 expression presents a significant challenge for preoperative biomarker assessment, as sampling limitations in fragmented biopsies may underestimate peak proliferative activity in biologically aggressive tumors. This is exemplified by this case, where the Ki67 index rose from 30% in the preoperative biopsy to 60% in the resection specimen, underscoring the potential unreliability of biomarker-guided decisions when relying solely on limited tissue samples.

Second, while emerging evidence supports anti-PD-1 agents in advanced mucosal melanoma (23), adjuvant immunotherapy was omitted here due to three key factors: the lack of consensus on its use in resected PARM, significant out-of-pocket costs exceeding $15,000 per cycle, and the patient’s preference following thorough shared decision-making.

Finally, the absence of circulating tumor DNA monitoring highlights a critical surveillance gap; though ctDNA represents a promising modality for minimal residual disease detection and recurrence prediction (24), its clinical utility requires prospective validation in dedicated PARM-specific protocols before routine implementation.

Importantly, while biomarkers such as elevated Ki67 indicate biological aggressiveness, their direct translation to clinical management decisions requires careful consideration. The current literature lacks direct comparisons of survival outcomes between radical resection and wide local excision specifically in PARM patients with high Ki67 indices. Future studies should aim to correlate biomarker profiles with long-term clinical outcomes to better define which patients truly benefit from more aggressive surgical approaches, particularly given the potential for increased morbidity with radical resection.

## 4 Conclusion

This S100-negative primary anorectal melanoma case underscores the importance of integrating biomarker profiling with conventional staging in PARM management. We demonstrate that a comprehensive immunohistochemical panel including SOX10, HMB45, and Melan-A is essential for accurate diagnosis of S100-negative cases. Furthermore, the preoperative Ki67 index of 30%, which increased to 60% in the resection specimen, provided critical biological information that supported the decision for radical resection despite radiologically localized disease.

While meta-analyses have suggested that wide local excision may offer comparable survival outcomes with lower morbidity compared to APR in selected PARM cases, our experience highlights that high-risk biological features such as markedly elevated Ki67 may identify a subset of patients who benefit from more aggressive local control. The absence of recurrence at 24-month follow-up in this patient with multiple high-risk features provides preliminary support for this biology-driven approach, though larger prospective studies are needed for validation.

For BRAF/NRAS wild-type PARM where immunotherapy access is limited, adjuvant temozolomide-cisplatin remains a clinically viable consideration, supported by prospective survival data in mucosal melanoma.

Moving forward, prospective validation of Ki67-guided surgical algorithms is critically needed to refine patient selection criteria. Such studies should delineate thresholds for escalating to radical resection versus permitting organ-preserving approaches, ultimately personalizing therapeutic intensity based on both tumor biology and anatomical staging.

## Data Availability

The original contributions presented in the study are included in the article/**Supplementary Material**. Further inquiries can be directed to the corresponding author/s.

## Supplementary material

Supplementary Figure 1 Histopathological examination of the resection specimen (H&E stain). **(A)** Low-power view (×20) showing the architecture. **(B)** High-power view (×100) revealing malignant cells with pleomorphic nuclei, prominent nucleoli, and cytoplasmic melanin deposition.
