# Supplementary figures and images for: Case Report: S100-negative primary anorectal melanoma with elevated Ki67: a case for biology-driven radical resection despite early radiological staging
Source: Front Oncol. 2025 Dec 11;15:1682861. doi: 10.3389/fonc.2025.1682861 (PMC12738296; doi:10.3389/fonc.2025.1682861)

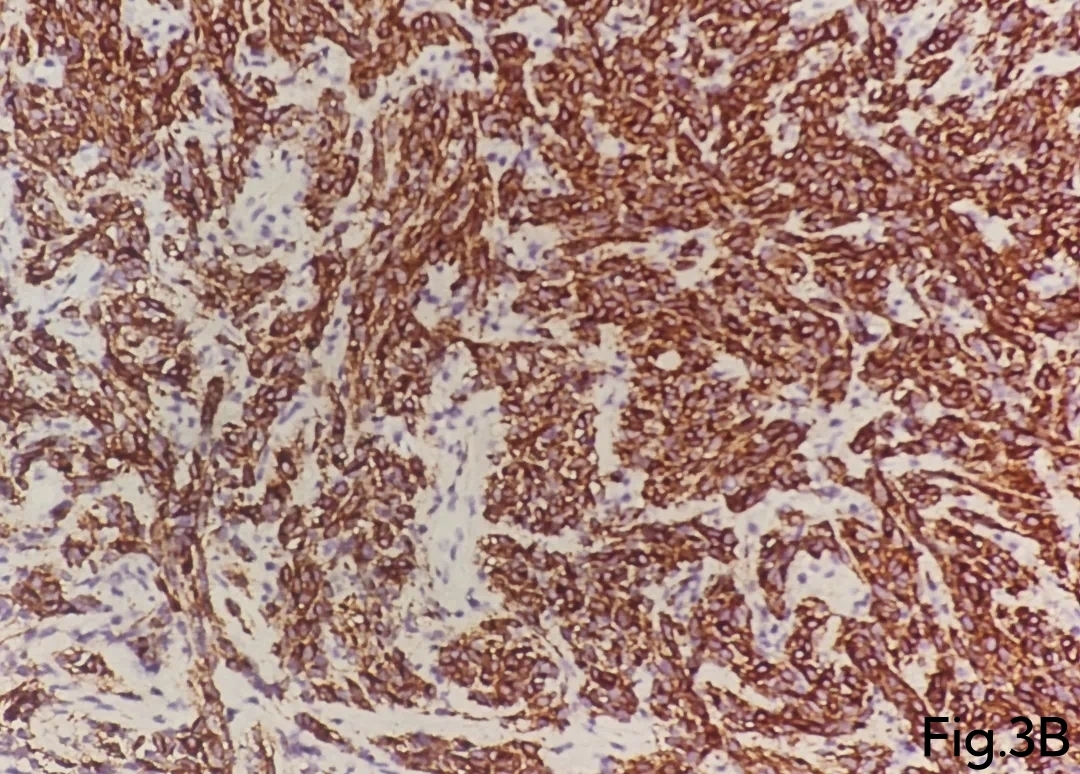

Supplement: Supplementary Figure 1 — Histopathological examination of the resection specimen (H&E stain). (A) Low-power view (×20) showing the architecture. (B) High-power view (×100) revealing malignant cells with pleomorphic nuclei, prominent nucleoli, and cytoplasmic melanin deposition. [file Image1.jpeg]

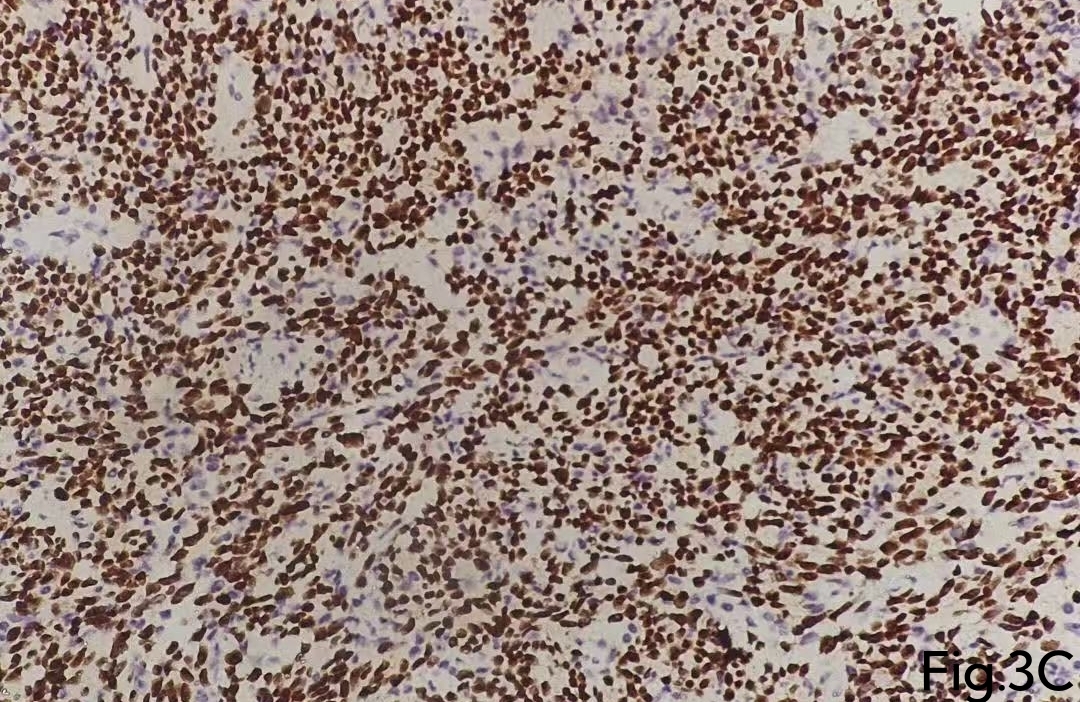

Supplement: Supplementary file 2 [file Image2.jpeg]

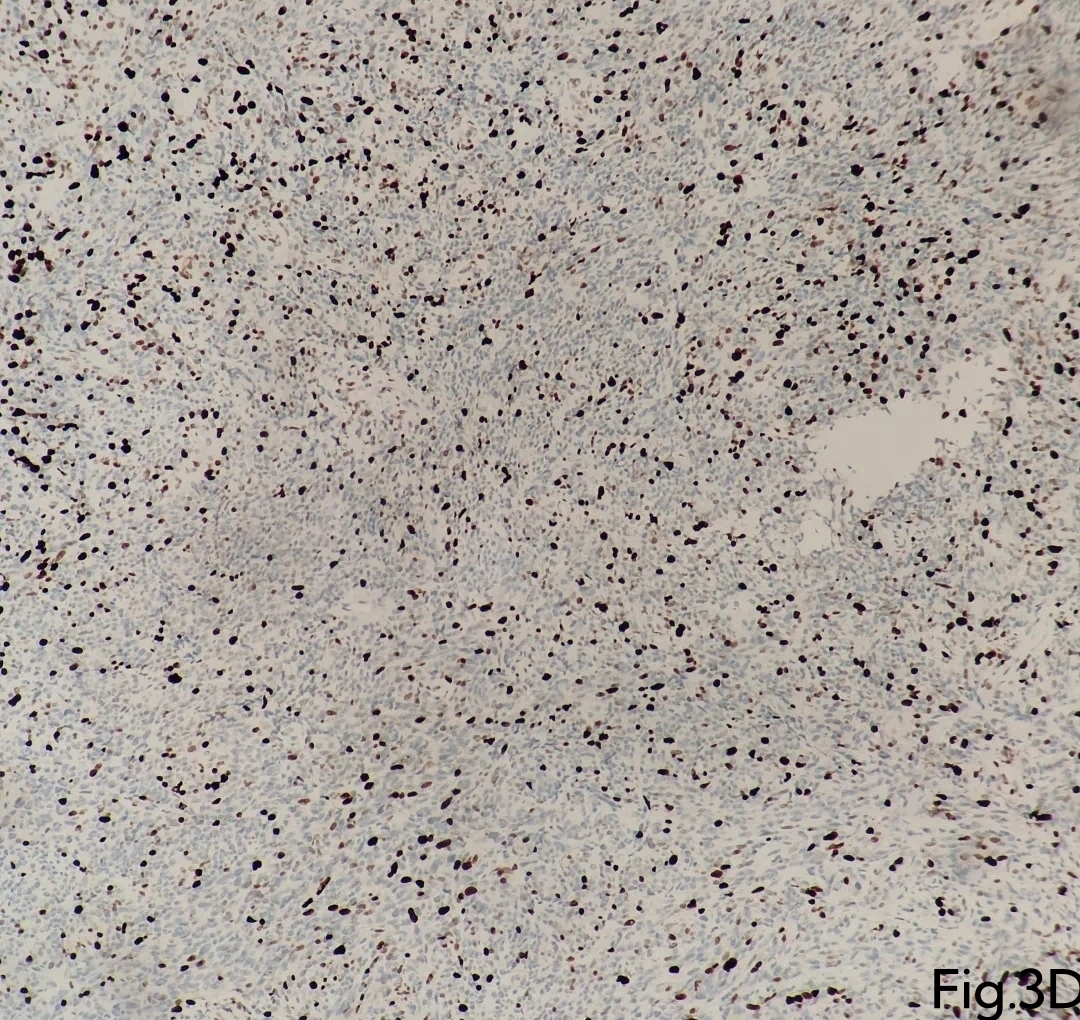

Supplement: Supplementary file 3 [file Image3.jpeg]

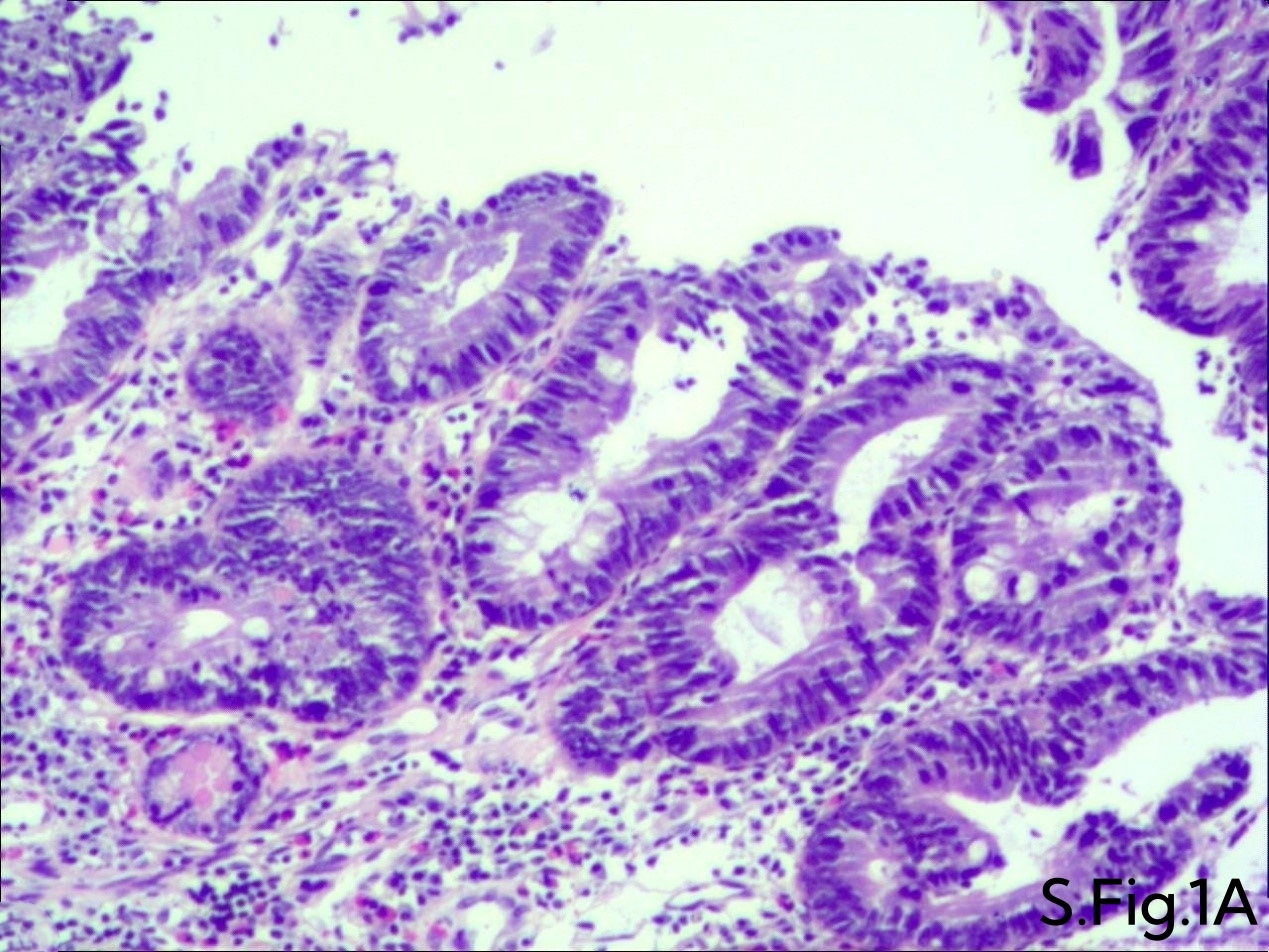

Supplement: Supplementary file 4 [file Image4.jpeg]

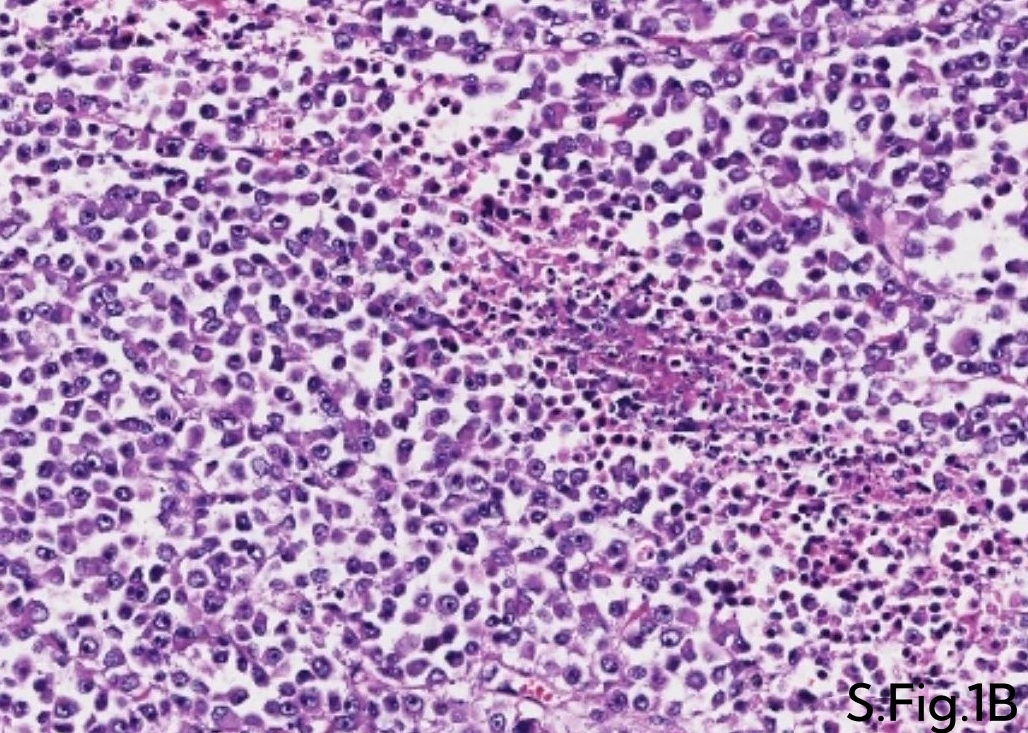

Supplement: Supplementary file 5 [file Image5.jpeg]
